# An evaluation of the discriminant and predictive validity of relative social disadvantage as screening criteria for priority access to public general dental care, in Australia

**DOI:** 10.1186/1472-6963-14-106

**Published:** 2014-03-04

**Authors:** Kelly Jones

**Affiliations:** 1 Australian Research Centre for Population Oral Health (ARCPOH), Dental School, Faculty of Health Sciences, The University of Adelaide, Adelaide, South Australia 5005, Australia

**Keywords:** Priority setting, Social disadvantage, Oral health, Predictive value, ROC analysis

## Abstract

**Background:**

Most public dental care services provide preventive, general dental care on a chronological, first come – first served basis. There is concern about lack of transparency, equity and timeliness in access to public dental services across Australia. Using social determinants as screening criteria is a novel approach to triage in dental care and is relatively untested in the literature. The research evaluated the discriminant and predictive validity of relative social disadvantage in prioritising access to public general dental care.

**Methods:**

A consecutive sample of 615 adults seeking general dental care was selected. The validation measure used was clinical assessment of priority. Nine indicators of relative social disadvantage (RSD) were collected: Indigenous status; intellectual disability; physical disability; wheelchair usage; dwelling conditions; serious medical condition; serious medical condition and taking regular medication; hospitalised within 12 months; and, regular medical visits. At the first dental visit, dentists rated care as a priority if treatment was required ≤6 months (PriorityTx) and otherwise non-priority (non-PriorityTx). A standardised dental examination was conducted. Sensitivity, specificity, positive and negative predictive value and area under the ROC curve analyses of 1+ of RSD in predicting clinical priority were calculated.

**Results:**

In bivariate analyses, one or more indicators of relative social disadvantage status were significantly associated with PriorityTx (P < 0.001; χ^2^). In multivariate analyses, one or more indicators of relative social disadvantage persisted as an independent predictor of PriorityTx (OR 3.8, 95% CI = 2.6-5.6). Compared with clinicians’ classification of PriorityTx, one or more indicators of relative social disadvantage had a sensitivity of 77.1%, and specificity of 53.3%, together with a positive predictive value of 81.9% and negative predictive value of 46.0%. ROC curve analysis supported one or more indicators of relative social disadvantage as a predictor of greater priority for access to general dental care (0.66).

**Conclusions:**

Considerable heterogeneity exists among persons seeking public general dental care in New South Wales. RSD performs as a valid predictor of priority for access to treatment and acts as valid screening criteria for triaging priority access to treatment. Such indicators may address issues of inequality in access to general public oral health services.

## Background

High demand for public dental services in Australia is stimulating the development of new approaches to prioritise access to public dental care. The New South Wales (NSW) Oral Health Branch in Australia has implemented a priority dental system, the Priority Oral Health Program (POHP), which replaced the traditional system of chronological queuing for access to public general dental care. POHP is a system for scheduling dental appointments, for both acute and general dental care. In the absence of acute need, POHP uses the presence of one or more indicators of relative social disadvantage (RSD) as a screening criterion to determine greater priority of patient access to general dental care. An expert Priority Oral Health Project Committee (comprising senior Area dental officers, dental specialists, dental policy makers and the then NSW Chief Dental Officer) developed the indicators and criteria for access to priority and non-priority general dental care.

The lack of a needs-based approach to managing dental treatment had raised concerns regarding both the fairness and timeliness of access to public oral health care in Australia, including NSW. In 1999, as a result of the very high demand for acute care, there was a growing perception that access to general care was being managed inequitably. There were anecdotal reports that due to work pressures and patient demands, staff responsible for scheduling appointments were assigning priority for general care subjectively, resulting in unfair variations in patient access and wait times between and within clinics. This was thought to reinforce the real or perceived inequity of the historically based, demand driven system. As such, it was believed that a systematic, uniform and transparent approach to patient access would be more equitable. Additionally, such an approach was perceived to potentially engender greater system efficiency through pooling all clinic waiting lists and also by removing the stress of clinical ‘gate keeping’ responsibilities faced by non-clinical reception staff [1,2] The subsequent introduction of POHP was part of a broader series of oral health reforms introduced simultaneously at the time, incorporating progressive funding increases, improved allocative and technical efficiencies, and performance monitoring, together with centralised “call centers” and a centralized management information system to manage the new system for access to dental care. However, these broader public policy issues will not be discussed here.

Two substantial issues stand out in such a priority system. First, the process of systematic use of hierarchical criteria based on self-report to explicitly ration access to general dental care. Such an approach challenged the role of clinicians in determining patients’ urgency and is both controversial and novel in the context of public health dentistry in Australia. However, subsequent anecdotal reports suggest that POHP is now a well-accepted patient management strategy. Priority setting using self reported symptoms in other areas of health care, such as orthopaedics and psychiatry, has long been an accepted strategy [3,4]. When first introduced in NSW however, POHP was contentious among clinical staff due to its use of a non clinical ranking system for access to general dental care [3,5].

Second, the use of social determinants as indicator variables for such prioritising is based in the hypothesis of greater acuity of clinical need among indigent populations when compared with more socially advantaged groups [6,7]. Subsequently, greater priority is allocated to individuals from population subgroups where both international and national research shows the existence of greater burden of disease when compared to the total population [8-13]. Such ‘positive discrimination’ is an attempt to introduce a more equitable system of access to care by rationing in the absence of a clinical assessment [14].

Ideally, screening tests are comprised of variables selected quantitatively *prior* to implementation and evaluation [2,3,15-17] However, as indicators of RSD in the NSW system were not selected by such means, their capacity in predictive classification of relative priority was not estimated prior to state-wide implementation. However, an evaluation process was incorporated into the state roll out of POHP. The research issue faced was whether such a heuristic screening model was clinically valid. This study reports on the evaluation of the discriminant and predictive validity of the POHP screening criteria for priority access to general public dental care.

## Methods

### POHP- an operational overview

POHP triages patients seeking emergency or relief of pain and general dental care. When a patient calls for care they are asked by reception staff an open-ended question “why have you contacted the clinic today?” Patient responses are checked against a list of possible dentally related answers with no prompts for response and answers catergorised accordingly. They are then recorded and processed by an integrated management information system (MIS), linking POHP prioritisation to appointment and even individual clinic and clinician scheduling. All patients are also asked a series of socio-demographic questions, including RSD questions. The MIS then uses patients’ responses and answers to calculate a patient’s relative priority and make an appointment for treatment. If, whilst waiting for general treatment, a patient’s condition worsens, they are able to recontact the clinic and complete the screening tool once again citing new or changed symptoms. Potentially patients can then be triaged into a more acute priority stream.

Patients, who did not report any emergency or relief of pain need, were considered to be eligible for general dental treatment. Any participants reporting an emergency need, such as relief of trauma, haemorrhage, swelling, dentally related pain, or medically compromised patients with a doctor’s referral were automatically excluded. Such patients were transferred into a parallel priority dental treatment triage stream, guaranteeing a “fast track” access to clinical assessment, between 24 hours and 10 days depending on their condition together with appropriate care. As such these patients were not included in this validation sample.

Allocated waiting times for patients seeking general treatment who reported one or more indicators of relative social disadvantage and patients reporting no RSD indicators differ. The maximum wait time allocated under POHP for persons with general treatment needs *and* one or more indicators of relative social disadvantage is 6 months with a minimum wait time of 1 month. For persons reporting general dental criteria and no RSD, the minimum waiting time is 7 or more months with no threshold on waiting time defined. A POHP expert committee determined these waiting times. Unequivocal clinically acceptable wait times for treatment for any general dental condition have not yet been reported in the literature.

### Ethics

Ethics approval was granted by the Human Research Ethics Committee of the Waiting Time Management Committee of the NSW Department of Health. Participants gave written informed consent before participating.

### Participants

This study used a consecutive sample of adult (18+ years of age) community dental patients holding a current government concession card who contacted clinics requesting general dental treatment during a maximum recruitment period of 12 months across 1999–2000. Six community dental clinics in New South Wales, Australia were selected to participate, of which three were metropolitan and three were rural clinics. Clinics were selected to maximise potential responses and subsequently selected on size. All patients contacting these clinics who reported at least one of the eligible clinical criteria for general dental treatment were recruited for the validation study until the required sample size was achieved. Of the 1200 participants desired, 1006 were recruited of which 610 had matching validation instruments. This resulted in a total sampling yield of 60.6% of the desired sample size.

### Data collection

Two types of self-reported data were utilised in the validation; the primary self-reported data was derived from the POHP triage screen comprising the indicator questions of relative social disadvantage and self-reported general dental screening criteria used. Reception staff were trained in the delivery of the screening tool and administered it when patients telephoned, or presented at the clinic, requesting dental treatment.

The General treatment screening criteria for priority for general dental treatment was assessed by asking the following question “Why have you contacted the clinic today?” The list of all possible responses is follows:

I have a broken filling

I need a filling

I have bleeding gums

I have a loose tooth

I have a broken tooth

I need an extraction

I have a chipped tooth

I have sore gums

I need a scale and clean

I have mouth ulcers

I need gum treatment

I have a broken denture

My denture needs to be fixed

I have lost my denture

I have a clicking jaw

I have Halitosis (bad breath)

I need a crown and/or bridge

1+ RSD Criteria-additional to clinical criteria “Are you /do you..?”

Aboriginal/Torres Straight Islander

Have a physical disability

Use a wheelchair

Have a Serious Medical Condition

Have a Serious Medical Condition and take regular medication

See a doctor regularly

Hospitalised in last 12 months

Have an intellectual disability

A boarding house resident/are homeless/institutionalised/caravan/hostel resident

Patients could report more than one criterion and the response format was a dichotomous Yes/No.

Secondary self-reported data instruments used were the shortened Oral Health Impact Profile (OHIP-14) questionnaire to evaluate patients reported impact on quality of life resulting from their dental condition and a standardised oral epidemiological examination was conducted from which index scores on oral health status were derived [18].

All study patients were fast tracked through the public dental system to minimise possible time related changes to their clinical dental condition. At the dental appointment, the examining dentist recorded a normative assessment of patient priority of access to treatment as an estimated number of months that could reasonably be waited until dental treatment. Dentists conducting the standardised dental examinations were not calibrated and assessed patient priority under the conditions of a routine clinical assessment. Dentists were blind to the RSD/ no RSD status of patients. However, indicators for RSD included physical criteria or Indigenous status, which may have been either apparent or would be disclosed during routine medical history taken during such a general treatment session.

#### Statistical and analytic methods

The dependent variable used for the validation was the dentist’s normative assessment of patients’ priority of access to treatment and was dichotomised by months to be waited before treatment into ≤6 months (PriorityTx) or 7+ months (no-PriorityTx).

Independent predictor variables came from the general dental treatment screening tool (see list of responses). A positive response to one or more indicators of relative social disadvantage from patients was used by the screening system to generate priority of access to general dental treatment. Multiple responses to RSD indicator questions were not allocated greater weighting by the MIS than a response to any one RSD indicator alone. Hence, a positive response of 1 or more of any of the indicator questions on RSD derived from the screening tool was collapsed into a single variable of one or more indicators of relative social disadvantage (1+ RSD) for analysis.

Bi-variate analysis of associations between the dependent and independent variables were performed. The Chi-square test was used to study the statistical significance of associations between the dependent variable and the 1 + RSD and general dental screening variables. Two-tailed *P* values less than 0.05 were considered significant.

To validate the priority system, significant association between one or more indicators of relative social disadvantage (1+ RSD) and dental assessment of priority of access to treatment (PriorityTx or no-PriorityTx) was required to measure the predictive capacity of 1+ RSD as a screening tool.

Logistic regression models were then used to test the predictive capacity of 1+ RSD as a screening tool. A univariate logistic regression model was fitted to the data to analyse the effect of the main independent variable of interest (1+ RSD) on the dependent variable of priority access to treatment ≤6 months (PriorityTx) or 7 + months (no-PriorityTx) [17,19]. The primary outcome measure is the predictive power of 1+ RSD as measured by Receiver Operator Characteristic (ROC) analysis. Further logistic regression modelling was performed to determine whether the predictive power of the screening test of 1 + RSD could be improved through the introduction of other independent variables collected from the general dental screening tool and statistical comparisons between the area under ROC performed [19,20]. Stata Statistical Software: Release 8.0 was used for the analysis [21].

## Results

### Sample characteristics

Persons reporting one or more indicators of relative social disadvantage were more likely to be male and were significantly older than persons not reporting any indicators of relative social disadvantage (P < 0.001; χ^2^). There was a significant difference between mean number of decayed teeth present, with persons with No RSD having higher mean decay scores but no difference between the mean number of missing or restored teeth and periodontal health scores between the two groups. See Table 1. Persons with one or more indicators of relative social disadvantage reported greater negative impact on their quality of life resulting from their current dental conditions as measured by mean total OHIP-14 score (P < 0.001; ANOVA).

**Table 1:** Mean oral health summary measure scores between persons reporting 1 + RSD and No RSD

	Decayed (D)*	**Filled (F)**	**Extracted due to caries (E)**	No. teeth present (T)**	**D/T ratio**	**Periodontal index score**
**1+ RSD**	**1.87**	**7.87**	**5.23**	**18.10**	**0.12**	**1.58**
**No RSD**	**2.41**	**9.11**	**4.49**	**23.19**	**0.13**	**1.68**

*ANOVA (P < 0.05).

**ANOVA(P < 0.01).

Chi-square analysis showed that 81.9% of persons reporting one or more indicators of relative social disadvantage were classified as needing PriorityTx (require treatment within 6 months) compared to 54.0% of those not reporting any indicator of RSD (P < 0.001; χ^2^).

When assessing the predictive validity of 1+ RSD as a screening tool, using the statistics for determining the strength of such a test, the sensitivity for one or more indicators of relative social disadvantage was 0.771. This indicates that 77.1% of those requiring priority treatment were identified by the screening tool as needing priority treatment. Specificity of 0.533 indicated 53.3% of the true non-priority cases were identified as non-priority by the screening tool. The positive predictive value of one or more indicators of relative social disadvantage as a screening tool indicates that the likelihood that a patient reporting one or more indicators of relative social disadvantage requires priority access to treatment than someone not reporting one or more indicators of relative social disadvantage is 81.9%. See Table 2.

**Table 2:** Predictive value of one or more indicators of relative social disadvantage for priority access to public general dental treatment

		**Dental assessment (observed)**	
		**PriorityTx % (n)**	**Non priorityTx % (n)**
Predicted	**1+ RSD**	81.9	18.1
		(345)	(76)
	**No- RSD**	54.0	46.0
		(102)	(87)

P < 0.0001; χ^2^ test. Sensitivity 77.1%, Specificity 53.3%, Positive Predictive Value 81.9% (95% CI, 0.77-0.85) Negative Predictive Value 46.0% (95% CI, 0.39-0.53), Positive Likelihood Ratio 1.65, Negative Likelihood Ratio 0.48.

In addition to persons reporting 1 + RSD, persons perceiving a need for a filling, having a broken filling, having a broken denture, needing an extraction and those not reporting needing a scale and clean were significantly more likely to be categorised by the dentist as PriorityTx; (P < 0.05; χ^2^). See Table 3.

**Table 3:** Proportions of self reported general care treatment or oral health need within persons categorised as PriorityTx

	**N**	**Response†**	**PriorityTx (row%)**
**General Treatment Screening Questions†**			
I have a broken filling*	83	Yes	63.9
I need a filling*	174	Yes	67.8
I have bleeding gums	7	Yes	42.9
I have a loose tooth	10	Yes	70.0
I have a broken tooth	83	Yes	67.5
I need an extraction*	45	Yes	86.7
I have a chipped tooth	20	Yes	60.0
I have sore gums	18	Yes	61.1
I need a scale and clean**	85	Yes	50.6
I have mouth ulcers	6	Yes	66.7
I need gum treatment	10	Yes	60.0
I have a broken denture**	46	Yes	91.3
My denture needs to be fixed	112	Yes	79.5
I have lost my denture	7	Yes	100.0
I have a clicking jaw	-	Yes	-
I have Halitosis (bad breath)	5	Yes	60.0
I need a crown and/or bridge	10	Yes	60.0
1+ RSD***	425	Yes	81.9
No RSD	190	Yes	54.0

†Response format: Self reported criterion. Patients could report multiple criterion.

***(P < 0.001), **(P < 0.01), *( P < 0.05); χ^2^.

### Regression analysis-Model 1

The nine variables that comprise one or more indicators of relative social disadvantage were collapsed into one variable (1 + RSD) and this was used in an unconditional regression model to determine whether one or more indicators of relative social disadvantage was indeed a significant predictor for general treatment PriorityTx see Table 3.

1 + RSD patients had 3.8 (CI 2.6-5.6) times the odds of being classified as requiring general treatment PriorityTx than general care patients not reporting any indicators of RSD (P < 0.001).

### Receiver operator characteristics (ROC) – Model 1

**Figure 1:**
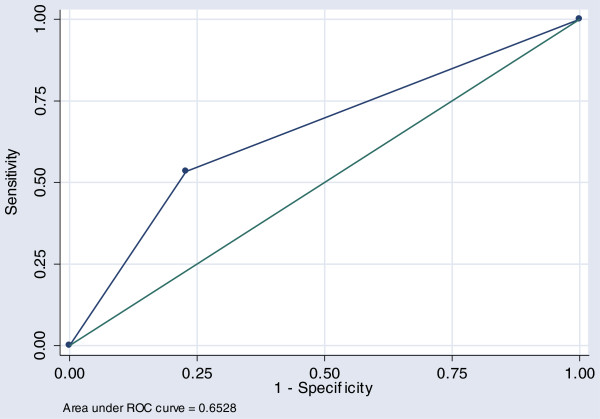
Analysis of Model 1.

ROC regression modeling was done to retrospectively measure the overall predictive performance of the screening tool; i.e.: one or more indicators of relative social disadvantage. The area under the ROC curve (AUR) is a measure of the accuracy, or the discriminative capacity of the screening test and ROC curve analyses shows the trade off between the differences in sensitivity and specificity of a test. A diagonal reference line (AUR = 0.50) defines points where a test is no better than chance in identifying priority individuals. The AUR from the univariate regression model using only one or more indicators of relative social disadvantage as an independent variable indicates that in 65.2% of all occasions a subject who requires priority treatment will have 1 + RSD characteristics than someone who is not classified as Non PriorityTx see Figure 1.

### Regression analysis*-*Model 2

To determine whether a more predictive model could be developed, regression modeling was performed using all general treatment screening questions, controlling for sex and age, in addition to one or more indicators of relative social disadvantage. Table 4 shows the odds ratios generated from a logistic regression of independent variables in predicting PriorityTx. The model shows that one or more indicators of relative social disadvantage, reporting needing an extraction and *not* reporting needing a scale and clean are significant predictors of PriorityTx.

**Table 4:** Unconditional regression model for those requiring treatment < 6 months using 1 + RSD and logistic regression model for those requiring treatment < 6 months using self-reported oral condition screening criteria and 1 + RSD

**Criteria**	**Beta coefficient**	**SE**	**Sig z**	**OR**
**Model 1**				
1 + RSD	1.35	0.19	0.000	3.87 (2.65, 5.65)
I have sore gums	−0.113	0.529	0.848	0.89 (0.27, 2.85)
I need a scale and clean	−1.156	0.086	0.000	0.31 (0.18, 0.53)
I have mouth ulcers	−0.013	1.067	0.990	0.98 (0.11,8.22)
I need gum treatment	0.412	1.182	0.598	1.51 (0.32,7.00)
I have a broken denture	1.001	1.540	0.077	2.72 (0.89,8.25)
My denture needs to be fixed	−0.121	0.257	0.676	0.88 (0.50,1.56)
I have Halitosis (bad breath)	−0.119	0.944	0.911	0.88 (0.11,7.14)
**Model 2**				
1 + RSD	1.302	0.827	0.000	3.67 (2.36,5.71)
I have a broken filling	−0.234	0.217	0.393	0.79 (0.46,1.35)
I need a filling	0.141	0.276	0.555	1.15 (0.72,1.84)
I have bleeding gums	−0.495	0.546	0.581	0.60 (0.10,3.53)
I have a loose tooth	0.291	0.989	0.693	1.33 (0.31,5.69)
I have a broken tooth	−0.049	0.270	0.862	0.95 (0.54, 1.66)
I need an extraction	1.370	1.854	0.004	3.93 (1.56, 9.91)
I have a chipped tooth	−0.263	0.379	0.593	0.76 (0.29,2.02)

Sensitivity 81.5%, Specificity 63.4%, Positive Predictive Value 87.2%.

Negative Predictive Value 52.8% (95% CI,) Cut off set at 0.7.

Criteria of having a lost denture, a clicking jaw and a perceived need for a crown and bridge have been dropped from the model due to colllinearity.

### Receiver operator characteristics (ROC) - Model 2

**Figure 2:**
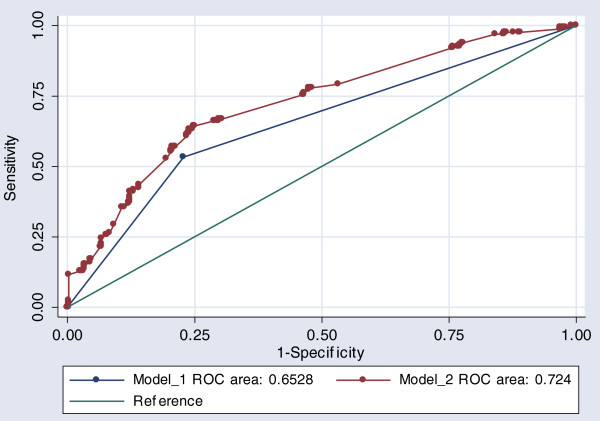
Comparison of AUR between Model 1 and Model 2.

Model 2 shows an AUR from the binary regression model of 72.3% see Figure 2. An AUR between 0.7-0.8 indicates ‘good’ predictive capacity and one or more indicators of relative social disadvantage in addition to other covariates from the general dental criteria, as a screening tool lies within this interval. Such a result suggests that one or more indicators of relative social disadvantage is a clinically rational and independent socio-demographic modifier to self-reported treatment need. The model suggests also that a more accurate and hence potentially equitable screening can be developed from the available criteria if relative weightings were introduced into priority calculations. Testing for a significant difference between the Model 1 AUR and Model 2 AUR shows that Model 2 performs significantly better than Model 1 in discriminating between persons requiring access to priority general treatment (P < 0.001; χ^2^) see Figure 2.

## Discussion

The pragmatic approach of the NSW Oral Health Branch, to introduce a heuristically developed priority system for triaging patients for access to general dental treatment has been shown in this study to be a valid one. Recognition of the role of socio-demographic characteristics as potential predictive covariates in determining an individuals’ priority for access to treatment and implementation of dental screening based on such characteristics is an example of a broader NSW Health population health strategy aimed at addressing inequalities and access to health. Arguably, since it is difficult for health services to make changes to these social determinants of health, NSW Health, incorporating NSW Oral Health, has aimed to reduce the impact of social disadvantage in *access* to healthcare.

The assumption of a lack of homogeneity between patients seeking public general dental treatment by NSW Oral Health Branch was a unique public health policy decision. Growing disparities in income levels, and increasing levels of social exclusion are causing increases in health inequalities in Australia. This validation study supports the hypothesis of a social gradient in such inequalities even within an already disadvantaged population [12,22].

Content and face validity of the POHP screening criteria were established prior to the implementation of POHP. Spectrum bias potentially driven by both patient and provider characteristics is perceived to have been minimised in this validation study due to both the sampling style employed and demographic and geographical variations between clinics selected [23,24]. The use of convenience sampling may raise some concerns about the implications of possible selection bias in the sample distribution. However, as all patients calling for treatment were consecutively selected, such concern may be unwarranted. Few standardised criteria for presentation and interpretation of oral symptoms exist and these are not determined by ‘gold standard’ measures against which judgment of either diagnosis and or treatment can be made [25]. As examiners were uncalibrated, the issue of verification bias remains unresolved given that there has always been a high level of discrepancy between dentist’s opinions in dentistry, as there is in most medical opinion where objective criterion or gold standard references do not exist [26-28].

The validity of the clinical assessment as the ‘gold standard’ measure on which the validation was based is a contentious one in dentistry. Such interpretation of patients’ oral health status and hence their relative priority attributed by practitioners, “operates in the absence of definitive diagnostic steps and contributes to the extensive variation among practitioners when they are asked to provide caries diagnosis or number and type of procedures and even teeth involved” [26,27,29-31]. However, the literature suggests that although low correlations between practitioners’ determination of disease presence, severity and treatment of individuals remains problematic in predicting treatment plans, such correlations may be useful in predicting resource supply or formulating broad public health policy decisions [13,32]. The application of epidemiological data in health services evaluation in an attempt to engender or validate evidence-based decision making for planning, administration and evaluation is relatively underdeveloped. However, as access to treatment is the issue under investigation in the POHP evaluation, and not the appropriateness of treatment subsequently received, then such statistical correlation at this level of analysis appears valid [26,27,33-36].

The lack of mutually exclusive indicator variables in defining 1 + RSD and need for general dental treatment has meant that issues of the potentially compounding nature of 2 or more characteristics of RSD and/or 2 or more general treatment screening criteria have not been built into the system. It is therefore recommended that further testing of weights derived from beta coefficient values from regression Model 2 and relative differences between 1 + RSD categories might prove to be useful in developing a more targeted or accurate model for screening [37]. Thus, it may increase the future sensitivity and specificity of the screening test and improve priority ranking of public dental patients.

The literature is unequivocal that RSD is a risk factor for poorer health outcomes and that indigent populations suffer greater morbidity and contribute disproportionately to the burden of disease estimates for chronic diseases [8,22,38]. This pragmatic policy decision is supported by and lends support to the concept of a social gradient in health; that the most deprived persons even within an already deprived population, have greater general health treatment needs [39]. Classification by clinical staff of greater priority of access to oral health care required by the most disadvantaged persons (those reporting 1 + RSD) and significantly higher mean scores of reported social impact suggest that such persons have greater normative need and that the subjective social impact experienced from their then current oral health status is greater for these persons.

## Conclusions

Various authors have explored the potential use of subjective oral health status measures in predicting oral health treatment need. While statistically significant associations between clinical indicators and subjective measures have been reported in the literature, the strength of associations between normatively assessed oral health need and subjective oral health status are not robust [32,39-43]. This validation study suggests that indicators of relative social disadvantage in conjunction with reported subjective oral health treatment need and oral health status can be used as a proxy measure for relative priority for access to general dental treatment. Such an outcome may be in part due to the fact that the population under observation in this validation was not asymptomatic and hence engendered greater discriminative and predictive power to the test [44]. Performance of the screening operates better than chance alone, or chronological queuing, which did not discriminate relative oral health need between patients seeking general dental care in NSW public dental clinics. Plans by NSW Oral health to further develop, test and incorporate relative weightings to generate greater allocate efficiency for access to general dental care and possibly better oral health outcomes, are supported by this validation.

## Competing interests

The author is an employee of ARCPOH at the University of Adelaide.

## Pre-publication history

The pre-publication history for this paper can be accessed here:

http://www.biomedcentral.com/1472-6963/14/106/prepub
